# Prevalence and incidence of heart failure in type 2 diabetes patients: results from a nationwide prospective cohort—the DIABET-IC study

**DOI:** 10.1186/s12933-024-02358-0

**Published:** 2024-07-16

**Authors:** Rafael Gonzalez-Manzanares, María Anguita-Gámez, Javier Muñiz, Vivencio Barrios, José Antonio Gimeno-Orna, Antonio Pérez, Luis Rodríguez-Padial, Manuel Anguita, on behalf of the DIABETIC-IC study Investigators

**Affiliations:** 1 Cardiology Unit, Reina Sofía University Hospital, Avda. Menéndez Pidal s/n, 14004 Córdoba, Spain; 2 Instituto Maimónides de Investigación Biomédica de Córdoba (IMIBIC), Córdoba, Spain; 3 Centro de Investigación Biomédica en Red Enfermedades Cardiovasculares (CIBERCV), Madrid, Spain; 4 Instituto Cardiovascular, Hospital Clínico San Carlos, Instituto de Investigación Sanitaria del Hospital Clínico San Carlos (IdSSC), Madrid, Spain; 5 Instituto Universitario de Ciencias de la Salud, Instituto de Investigación Biomédica de A Coruña (INIBIC), Universidade da Coruña, La Coruña, Spain; 6 Cardiology Department, University Hospital Ramon y Cajal, Madrid, Spain; 7 Endocrinology and Nutrition Department, Hospital Clínico Universitario Lozano Blesa, Zaragoza, Spain; 8 Centro de Investigación Biomédica en Red de Diabetes y Enfermedades Metabólicas Asociadas (CIBERDEM), Madrid, Spain; 9 Servicio de Endocrinología y Nutrición, Hospital de la Santa Creu i Sant Pau, Universitat Autònoma de Barcelona, Barcelona, Spain; 10 Cardiology Unit, Complejo Hospitalario de Toledo, Toledo, Spain

**Keywords:** Heart failure, Diabetes mellitus, Cardiovascular disease, Cardiometabolic

## Abstract

**Background:**

Type 2 diabetes (T2D) patients have an increased risk of heart failure (HF). There are limited data on the association between HF and T2D in specific healthcare settings. This study sought to analyse the prevalence and incidence of HF in a contemporary cohort of T2D patients attending cardiology and endocrinology outpatient clinics.

**Methods:**

We conducted an observational multicentre prospective study (DIABET-IC) that enrolled patients with a T2D diagnosis attending cardiology and endocrinology outpatient clinics in 30 centres in Spain between 2018 and 2019. The prevalence at the start of the study and the incidence of HF after a 3 year follow-up were calculated. HF was defined as the presence of typical symptoms and either: a) LVEF < 40%; or b) LVEF ≥ 40% with elevated natriuretic peptides and echocardiographic abnormalities.

**Results:**

A total of 1249 T2D patients were included in the present analysis (67.6 ± 10.1 years, 31.7% female). HF was present in 490 participants at baseline (prevalence 39.2%), 150 (30.6%) of whom had a preserved ejection fraction. The presence of adverse social determinants and chronic conditions such as chronic kidney disease and atherosclerotic cardiovascular disease were more frequent in HF patients. During the study period, there were 58 new diagnoses of HF (incidence 7.6%) among those without baseline HF. The incidence rate was 3.0 per 100 person-years. Independent predictors of incident HF were smoking, left ventricular ejection fraction, NT-ProBNP, history of tachyarrhythmia and treatment with pioglitazone, oral anticoagulants, or diuretics. Despite an average suboptimal glycaemic control, the use of antidiabetic drugs with cardiovascular benefits was low (30.4% for sodium-glucose cotransporter-2 inhibitors and 12.5% for glucagon-like peptide-1 receptor agonists).

**Conclusions:**

In this contemporary cohort of T2D patients attending cardiology and endocrinology outpatient clinics, the prevalence and incidence of HF were high, comorbidities were frequent, and the use of antidiabetic agents with cardiovascular benefit was low. Outpatient care seems to be a unique opportunity for a comprehensive T2D approach that encompasses HF prevention, diagnosis, and treatment.

**Graphical Abstract:**

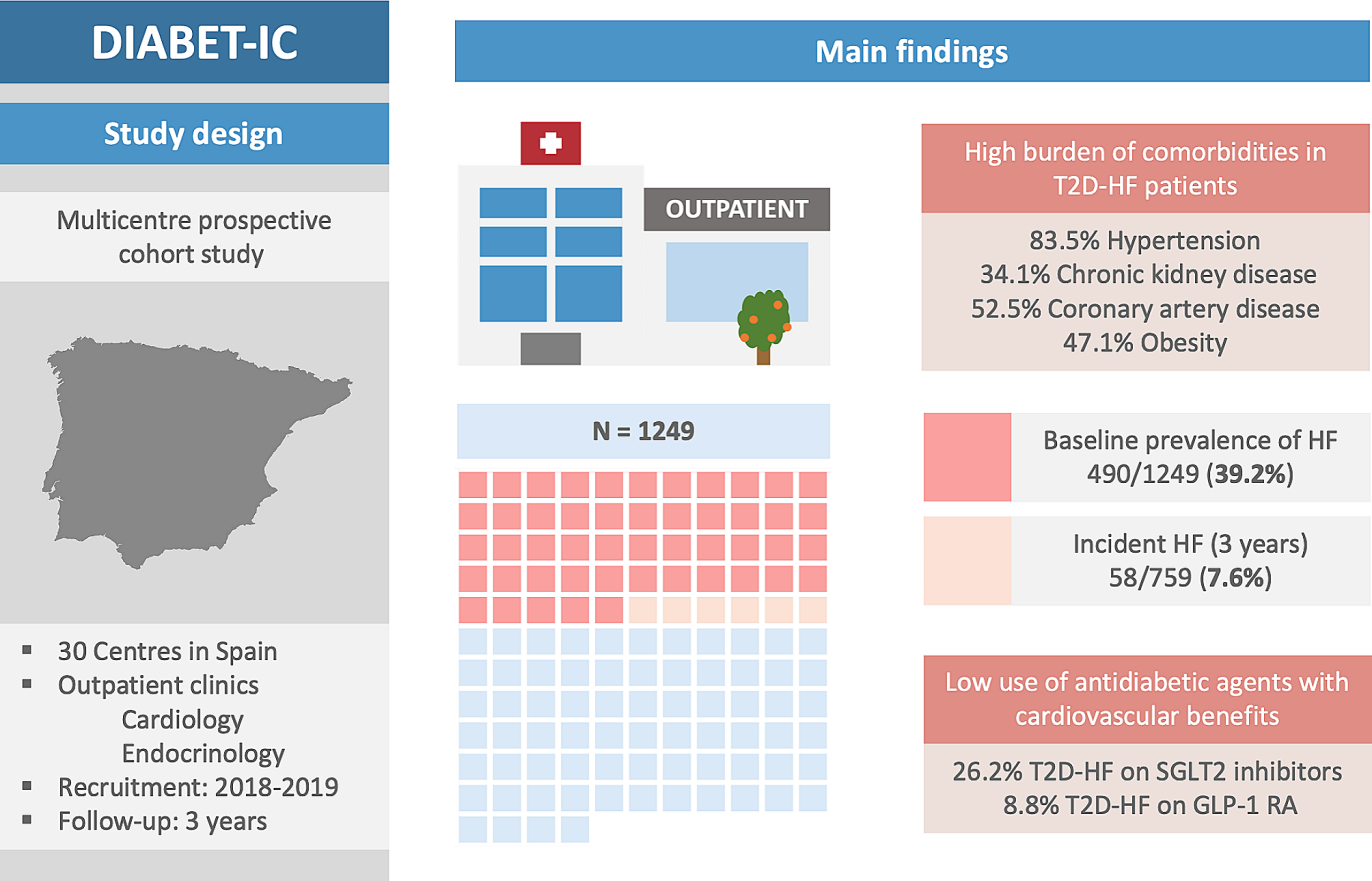

## Background

Heart failure (HF) is a major health problem that affects more than 50 million people worldwide, constituting a main cause of quality-of-life impairment and disability for individuals, and a major economic problem for health systems [1]. Up to 2% of healthcare budgets are allocated to HF, with most of the costs attributed to hospitalisation [2, 3]. The prevalence of HF and related hospitalisations has increased over the last decade, and this trend is expected to exacerbate because of the ageing population, posing a challenge for healthcare services [4]. Thus, adequate allocation of healthcare resources and the implementation of policies aimed at the prevention and early diagnosis of this condition are of utmost importance.

Type 2 diabetes (T2D) is a growing pandemic estimated to affect up to 529 million individuals globally, with a substantial impact on healthcare costs and patient morbimortality and quality of life [5, 6]. Although cardiovascular disease prevention and management of patients with T2D have traditionally focused on atherosclerotic cardiovascular disease, the bidirectional relationship between T2D and HF is receiving growing attention in light of the favourable effects of novel antidiabetic drugs on HF-related outcomes and quality of life [7–10]. Patients with T2D have 2 to 5 increased risks of HF and the prevalence of HF in these patients is reported to be up to 22% [11–13]. However, this association might be even greater under active surveillance using contemporary tools and updated diagnostic criteria for HF that enable diagnosis at initial stages.

The purpose of this study was to assess the prevalence and incidence of HF in a nationwide multicentre prospective cohort of T2D patients using contemporary diagnostic methods. Second, we aimed to describe the characteristics and predictors of incident HF in these patients.

## Methods

### Study design and setting

The DIABET-IC study is a multicentre, observational, and prospective cohort study carried out in 30 centres in Spain and promoted by the Spanish Society of Cardiology and the Spanish Society of Diabetes. The main aim was to evaluate the prevalence and incidence of HF in T2D patients visiting cardiology and endocrinology outpatient clinics in Spain. The baseline data of the cohort and sex-differences at presentation have been reported previously [14]. Enrolment of the study participants occurred between 2018 and 2019 and the follow-up time was 3 years. Recruitment was limited to the first 20 patients with a T2D diagnosis attending a designated cardiologist and endocrinologist clinics at each participating centre. Written informed consent was obtained from all the participants. The protocol was approved by the Clinical Research Ethics Committee of the University Hospital of Toledo (identification number 243) and the study was conducted in accordance with institutional and Good Clinical Practice guidelines. The current work follows the STROBE (STrengthening the Reporting of OBservational studies in Epidemiology) guidelines for reporting observational studies [15].

### Participants

Consecutive patients who attended cardiology and endocrinology outpatient clinics were considered eligible if they were aged 18 years or older and had a diagnosis of T2D at least one year prior to enrolment. The exclusion criteria were negative or inability to provide informed consent, end-stage chronic kidney disease, life expectancy inferior to three years due to cancer or other severe conditions and participation in an ongoing clinical trial. During the study design, it was expected that approximately half of the patients would be recruited from cardiology departments and the other half from endocrinology departments. However, due to slightly lower recruitment rates in the former, the final percentages were 61.9% and 38.1%, respectively.

The baseline visit consisted of a detailed medical history, physical examination, electrocardiogram, 2-dimensional echocardiography, and laboratory tests including N-terminal pro-B-type natriuretic peptide (NT-ProBNP) and glycosylated haemoglobin (HbA1c). After inclusion, periodic follow-up with at least a planned annual visit for 3 years was conducted. If HF was suspected by an endocrinologist, the cardiologist of that centre was responsible for making the definitive diagnosis and monitoring the confirmed cases throughout the study. The detailed criteria for HF diagnosis are specified in the next apart. All the participants received standard of care and enrolment did not imply additional interventions deviating from the routine practice management and treatment of HF and T2D.

### Variables, data sources and measurement

The following categories of variables were collected: demographic, comorbidities, HF and T2D treatment, echocardiographic and laboratory. T2D was defined according to the 2018 American Diabetes Association criteria [16]. HF was defined as the presence of a documented hospitalisation with a main diagnosis of HF during the previous year or the fulfilment of the 2016 European Society of Cardiology guidelines criteria for HF [17]. In brief, a HF diagnosis required the presence of typical symptoms plus additional echocardiographic and NT-proBNP abnormalities. For patients with HF with reduced ejection fraction (HFrEF), the presence of symptoms and a LVEF < 40% was enough. For patients with HF with mid-range ejection fraction HFmrEF (LVEF 40–49%) and HF with preserved ejection fraction HFpEF (LVEF ≥ 50%), both elevated levels of natriuretic peptides (NT-proBNP > 125 pg/mL) and functional (diastolic dysfunction) or structural (left ventricular hypertrophy or left atrial enlargement) abnormalities on echocardiogram were required. The HF diagnosis was confirmed in all cases by the reference cardiologist. Hypertension was defined as confirmed systolic blood pressure ≥ 140 mmHg, diastolic blood pressure (DBP) ≥ 90 mmHg, or use of antihypertensive medications. Dyslipidaemia was defined as use of lipid-lowering medications or total cholesterol levels > 240 mg/dl and/or LDL cholesterol > 160 mg/dl and/or triglycerides > 200 mg/dl and/or HDL cholesterol < 40 mg/dl in males or < 50 mg/dl in females. Chronic kidney disease was defined as a defined as the presence of an estimated glomerular filtration rate less than 60 ml/min /1.73 m^2^. Coronary artery disease was defined as documentation of acute myocardial infarction, acute coronary syndrome, coronary revascularization, or coronary stenosis > 50%. Peripheral artery disease was defined as the presence of lower extremity arteriopathy. Cerebrovascular disease was defined as documentation of ischemic or haemorrhagic stroke or carotid stenosis > 50%.

### Study size

A sample size of 1510 participants was calculated in the initial protocol. This calculation was based on the primary study hypothesis that the prevalence of HF in a contemporary cohort of T2D patients visiting cardiology and endocrinology outpatient clinics is significantly higher than previously reported rates. To ensure adequate power to detect an estimated prevalence rate of 30% at baseline and an incidence rate of 3% over a 3 year follow-up period, we made the following assumptions: a confidence level of 95%, a precision of 3%, and an anticipated loss to follow-up rate of 15%.

### Statistical methods

Categorical variables are presented as counts (percentages) and continuous variables are summarized as mean ± standard deviation or median (interquartile range) according to their distribution. Normality was assessed using the Shapiro–Wilk test and QQ plots. Between-group comparisons were performed using the chi-square test for categorical data and Student’s t-test or Wilkoxon rank-sum test for continuous data. Multivariable Cox regression was used to determine significant predictors for incident HF. The Cox model was built using backward stepwise elimination, initially including clinically relevant variables and those with a p < 0.100 in the univariable models. The proportional hazards assumption was assessed using Schoenfeld residuals. For all tests, a two-sided p < 0.05 was considered significant.

## Results

### Study population

**Fig. 1:**
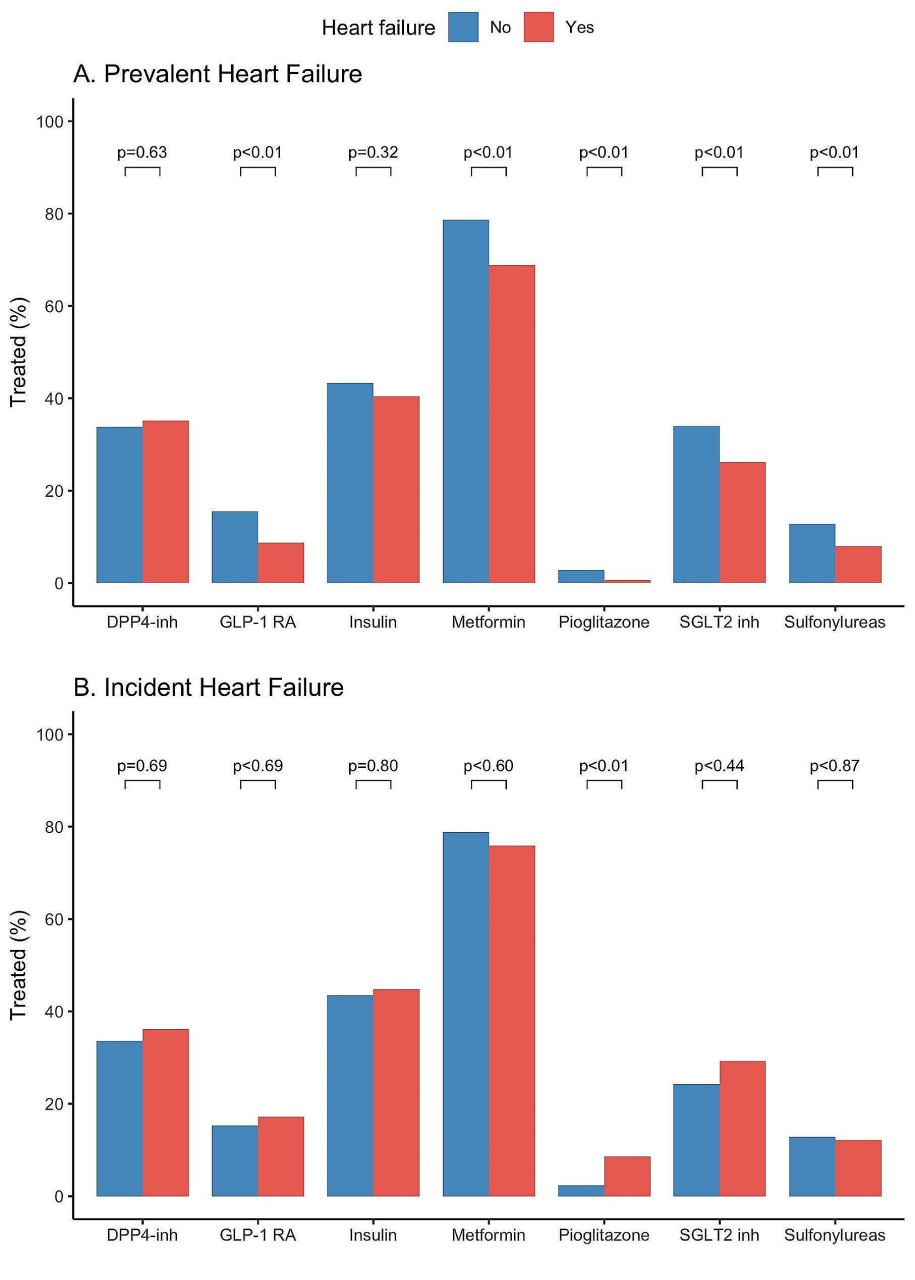
Antidiabetic therapies according to the diagnosis of heart failure at baseline (**A**) or follow-up (**B**). *DPP4-inh* Dipeptidyl peptidase-4 inhibitors, *GLP-1 RA* Glucagon-like peptide-1 receptor agonists, *SGLT2* inh Sodium-glucose cotransporter-2 inhibitors

A total of 1517 T2D patients attending cardiology and endocrinology outpatient clinics at 30 Spanish centres were enrolled between January 2018 and December 2019. Of them, 1249 patients with available follow-up data were included in the current analyses. The mean age was 67.6 ± 10.1 years and 31.7% were women. The mean duration of T2D was 6.8 ± 10.0 years. The prevalence of comorbidities other than T2D was high: hypertension (81.3%), dyslipidaemia (81.3%), chronic kidney disease (CKD) (23.5%), atrial fibrillation (AF) (23.0%), coronary artery disease (43.0%), peripheral artery disease (10.8%), cerebrovascular disease (8.2%), and obesity (47.6%). Most of the patients received metformin (73.6%) and one in three were treated with insulin. The prescription of antidiabetic drugs with direct cardiovascular benefits was low, with 30.4% of the participants receiving sodium-glucose cotransporter-2 (SGLT2) inhibitors and only 12.5% receiving glucagon-like peptide-1 (GLP-1) receptor agonists (Fig. 1). Half of the patients received diuretic treatment (51.4%).

### Prevalence of heart failure and comparison of the characteristics between HF and non-HF patients at the initial visit

The prevalence of HF at the initial visit was 39.2% (490 patients). Of these, 246 (50.2%) had HFrEF, 94 (19.2%) had HFmrEF, and 150 (30.6%) had a HFpEF. Of the 490 prevalent HF cases, 315 (64.3%) had a prior HF hospitalisation. There were 22 newly HF cases discovered through systematic screening at baseline: 5 (22.7%) with HFrEF, 6 (27.2%) with HFmrEF, and 11 (50%) with HFpEF. The characteristics of the participants grouped according to HF diagnosis at the initial visit are shown in Table 1. There were no sex differences between the groups. Conversely, HF patients were older, had lower levels of education, were less likely to be active workers, and were less frequently living with a partner. T2D complications such as diabetic nephropathy and diabetic foot were more frequent among HF patients. In half of the participants, HbA1c levels were above the recommended target: 7.0 (6.3–7.8) and 7.2 (6.5–7.9) %, in HF and non-HF patients, respectively (p = 0.073). There were significant differences in the burden of comorbidities between the two groups. Chronic obstructive pulmonary disease, sleep apnoea, CKD, peripheral artery disease, cerebrovascular disease, coronary heart disease and AF were more prevalent in the HF group. The average body mass index was high in both groups: 30.1 ± 5.6 vs. 30.2 ± 5.5 in HF and non-HF patients, respectively (p = 0.609). Among the laboratory results, c-LDL levels were slightly lower in the HF group, but these participants presented a more adverse lipid profile reflected by a higher triglyceride/c-HDL ratio (3.2 [2.3–4.9] vs. 2.9 [1.9–4.6], p < 0.001). NT-ProBNP levels were obviously higher in the HF group (784 [289–2090] vs 132[60–306] pg/mL, p < 0.001). With respect to the electrocardiogram findings, AF, pacemaker rhythm, and left bundle branch block were more frequent in the HF group. Conversely, right bundle branch block was more frequently reported among non-HF participants.

**Table 1:** Characteristics of the participants according to HF diagnosis at baseline

	No HF (n = 759)	HF (n = 490)	p
Age, years	66.0 ± 9.8	69.4 ± 9.8	< 0.001
Female sex	250 (32.9)	146 (29.8)	0.244
Employment status			< 0.001
Employed	207 (27.3)	51 (10.4)	
Unemployed	37 (4.9)	13 (2.6)	
Retired	419 (55.3)	337 (68.8)	
Disabled	16 (2.1)	45 (9.2)	
Housewife	79 (10.4)	44 (8.9)	
Family status			0.027
Lives with a partner	566 (74.9)	334 (68.2)	
Lives with relatives	100 (13.2)	95 (19.4)	
Lives alone	86 (11.4)	58 (11.8)	
Institutionalized	4 (0.5)	3 (0.6)	
Level of education			< 0.001
Illiterate	15 (1.9)	20 (4.1)	
Primary education	409 (54.1)	312 (63.7)	
Secondary education	183 (24.2)	105 (21.4)	
Vocational training	76 (10.1)	23 (4.7)	
University education	73 (9.7)	30 (6.1)	
SBP, mmHg	137.8 ± 18.4	129.8 ± 19.9	< 0.001
DBP, mmHg	76.9 ± 11.1	73.1 ± 11.4	< 0.001
BMI, Kg/m^2^	30.2 ± 5.5	30.2 ± 5.6	0.609
Obesity	368 (48.7)	227 (47.1)	0.587
Hypertension	606 (79.8)	509 (83.5)	0.109
Dyslipidemia	622 (81.9)	393 (80.2)	0.440
Smoking history			0.032
Never	355 (46.8)	205 (41.8)	
Former (< 1 year)	84 (11.1)	43 (8.8)	
Former (≥ 1 year)	21 (2.3)	24 (4.9)	
Current	299 (39.4)	218 (44.5)	
COPD	63 (8.3)	70 (14.3)	< 0.001
Chronic kidney disease	126 (16.6)	167 (34.1)	< 0.001
Cancer	51 (6.7)	44 (8.9)	0.141
Coronary artery disease	280 (37.0)	257 (52.5)	< 0.001
Cerebrovascular disease	52 (6.9)	50 (10.2)	0.036
Peripheral artery disease	61 (8.1)	74 (15.1)	< 0.001
Atrial fibrillation	89 (11.8)	187 (38.2)	< 0.001
Hemoglobin, g/dL	14.3 ± 1.7	13.7 ± 1.9	< 0.001
EGFR, ml/min/1.73 m^2^	78.7 ± 20.9	65.3 ± 23.4	< 0.001
Albuminuria, mg/g	11.8 (3.3–32.3)	10.0 (3.0–34.0)	0.392
Total cholesterol, mg/dL	156.2 ± 35.6	148.3 ± 36.5	0.001
LDL cholesterol, mg/dL	81.5 ± 29.6	77.5 ± 30.3	0.023
HDL cholesterol, mg/dL	45.5 ± 12.5	41.8 ± 11.8	< 0.001
Triglyceride, mg/dL	149.9 ± 83.2	155.9 ± 89.1	0.053
Triglyceride/HDL cholesterol	3.7 ± 2.9	4.2 ± 3.4	< 0.001
HbA1c, %	7.2 (6.5–7.9)	7 (6.3–7.8)	0.073
NT-ProBNP, pg/mL	132 (60–306)	784 (289–2090)	< 0.001
Rhythm (ECG)			< 0.001
Sinus rhythm	686 (90.4)	313 (63.9)	
Atrial fibrillation	58 (7.6)	124 (25.3)	
Pacemaker	11 (1.4)	43 (8.7)	
Other	4 (0.5)	10 (2.0)	
Conduction abnormalities (ECG)			< 0.001
Not present	653 (86.0)	371 (75.7)	
First-degree AV block	23 (3.0)	13 (2.7)	
RBBB	58 (7.6)	24 (4.9)	
LBBB	25 (3.3)	82 (16.7)	
LV hypertrophy (ECG)	290 (38.2)	184 (37.5)	0.791

Categorical variables are presented as n (%). Continuous variables are presented as mean ± standard deviation or mean (interquartile range)

*HF* heart failure, *SBP* systolic blood pressure, *DBP* diastolic blood pressure, *BMI* body mass index, *COPD* chronic obstructive pulmonary disease, *EGFR* estimated glomerular filtration rate, *LDL* low density lipoprotein, *HDL* high density lipoprotein, *HbA1c* Glycated hemoglobin, *NT-ProBNP* N-terminal pro-B-type natriuretic peptide. *ECG* electrocardiogram, *AV* atrioventricular, *RBBB* right bundle branch block, *LBBB* left bundle branch block

### Incidence of heart failure and comparison of the characteristics between HF patients and non-HF patients at follow-up

After a 3-year follow-up, new-onset HF was diagnosed in 58 of the 759 participants with no HF at baseline, resulting in an incidence of 7.6% and an incidence rate of 3.0 per 100 person-years. Of these, 14 (24.1%) were HFrEF, 17 (29.3%) were HFmrEF, and 27 (46.6%) HFpEF. The baseline characteristics of the participants, according to the occurrence of HF at follow-up and excluding those patients with HF at the initial visit, are shown in Table 2. In the unadjusted analysis, incident HF was not associated with age or sex but was more frequent in patients with lower education levels. Hypertension, CKD, and atherosclerotic cardiovascular diseases were not associated with incident HF. Conversely, a history of tachyarrhythmia was a predictor of HF. With respect to laboratory data, incident HF patients had higher baseline NT-ProBNP levels [220 (87–709) vs. 126 (56–296) pg/mL, p = 0.015) but comparable levels of HbA1c. In the Cox adjusted analysis, the significant predictors of incident HF were smoking, LVEF, NT-ProBNP, prior tachyarrhythmia and treatment with pioglitazone, oral anticoagulants, or diuretics (Table 3).

**Table 2:** Characteristics of the participants according to HF diagnosis at follow-up

	No HF (n = 701)	Incident HF (n = 58)	p
Age, years	65.9 ± 9.8	66.5 ± 9.9	0.594
Female sex	232 (33.1)	18 (31.0)	0.748
Employment status			0.391
Employed	190 (27.1)	17 (29.3)	
Unemployed	35 (5.0)	2 (3.5)	
Retired	384 (54.9)	35 (60.3)	
Disabled	14 (2.0)	2 (3.5)	
Housewife	77 (11.0)	2 (3.5)	
Family status			0.634
Lives with a partner	526 (75.4)	40 (69.0)	
Lives with relatives	90 (12.9)	10 (17.2)	
Lives alone	78 (11.2)	8 (13.8)	
Institutionalized	4 (0.6)	0 (0)	
Level of education			0.020
Illiterate	14 (2.0)	1 (1.7)	
Primary education	366 (52.4)	43 (74.1)	
Secondary education	175 (25.1)	8 (13.8)	
Vocational training	71 (10.2)	5 (8.6)	
University education	72 (10.3)	1 (1.7)	
SBP, mmHg	138.1 ± 18.4	133.8 ± 18.3	0.270
DBP, mmHg	77.2 ± 11.2	73.9 ± 10.5	0.083
BMI, Kg/m^2^	30.2 ± 5.6	30.8 ± 5.2	0.341
Obesity	334 (47.9)	34 (58.6)	0.114
Hypertension	558 (79.6)	48 (82.8)	0.565
Dyslipidemia	576 (82.1)	46 (79.3)	0.587
Smoking history			0.049
Never	330 (47.1)	25 (43.1)	
Former (< 1 year)	72 (10.3)	12 (20.7)	
Former (≥ 1 year)	18 (2.6)	3 (5.2)	
Current	281 (40.1)	18 (31.0)	
COPD	57 (8.1)	6 (10.3)	0.557
Chronic kidney disease	113 (16.1)	13 (22.4)	0.216
Cancer	45 (6.4)	6 (10.3)	0.251
Coronary artery disease	258 (37.0)	22 (37.8)	0.883
Cerebrovascular disease	47 (6.7)	5 (8.6)	0.583
Peripheral artery disease	54 (7.7)	7 (12.1)	0.243
Atrial fibrillation	78 (11.2)	11 (18.9)	0.077
Hemoglobin, g/dL	14.3 ± 1.7	14.1 ± 1.8	0.695
EGFR, ml/min/1.73 m^2^	78.7 ± 20.8	78.0 ± 23.4	0.783
Albuminuria, mg/g	11.3 (3.2–32.0)	12.9 (4.0–42.0)	0.489
Total cholesterol, mg/dL	156.9 ± 35.7	147.3 ± 33.8	0.032
LDL cholesterol, mg/dL	82.2 ± 29.4	73.1 ± 30.5	0.235
HDL cholesterol, mg/dL	45.6 ± 12.5	43.9 ± 12.4	< 0.001
Triglycerides, mg/dL	147.7 ± 79.7	176.3 ± 114.7	0.219
Triglycerides/HDL cholesterol	3.7 ± 2.9	4.6 ± 3.2	0.296
HbA1c, %	7.2 (6.5–7.9)	7.4 (6.2–8.2)	0.576
NT-ProBNP, pg/mL	126 (56–296)	220 (87–709)	0.015
Rhythm (ECG)			0.129
Sinus rhythm	638 (91.0)	48 (82.8)	
Atrial fibrillation	50 (7.1)	8 (13.8)	
Pacemaker	9 (1.2)	2 (3.5)	
Other	4 (0.6)	0 (0)	
Conduction abnormalities (ECG)			0.579
Not present	606 (86.4)	47 (81.0)	
First-degree AV block	20 (2.9)	3 (5.2)	
RBBB	53 (7.6)	5 (8.6)	
LBBB	22 (3.1)	3 (5.2)	
LV hypertrophy (ECG)	264 (37.7)	26 (44.8)	0.285

Categorical variables are presented as n (%). Continuous variables are presented as mean ± standard deviation or mean (interquartile range)

*HF* heart failure, *SBP* systolic blood pressure, *DBP* diastolic blood pressure, *BMI* body mass index, *COPD* chronic obstructive pulmonary disease, *EGFR* estimated glomerular filtration rate, *LDL* low density lipoprotein, *HDL* high density lipoprotein, *HbA1c* Glycated hemoglobin, *NT-ProBNP* N-terminal pro-B-type natriuretic peptide, *ECG*: electrocardiogram, *AV* atrioventricular, *RBBB* right bundle branch block, *LBBB* left bundle branch block

**Table 3:** Multivariable Cox regression for incident heart failure

	HR	CI 95%		p
LVEF, %	0.91	0.88	0.93	< 0.001
Smoking history	2.26	1.13	4.50	0.020
NT-ProBNP, pg/mL	1.01	1.00	1.01	< 0.001
Diuretics	2.78	1.45	5.33	0.002
Pioglitazone	3.62	1.30	10.02	0.013
SGLT2 inhibitors	0.69	0.40	1.19	0.188
Oral anticoagulant	2.82	1.38	5.76	0.004
History of tachyarrhythmia	2.49	1.11	5.61	0.027

*HR* Hazard ratio, *CI* confidence interval 95%, *LVEF* Left ventricular ejection fraction, *NT-ProBNP* N-terminal pro-B-type natriuretic peptide

### Clinical events

After a mean follow-up period of 2.6 years, 122 deaths were reported. Of these, 46 were attributed to cardiovascular causes: HF (n = 26), acute coronary syndrome (n = 6), stroke (n = 10), and sudden cardiac death (n = 4). HF hospitalization occurred in 30 patients. No patients received advanced heart failure therapies such as heart transplantation or left ventricular assist devices.

## Discussion

In this contemporary cohort of T2D patients visiting cardiology and endocrinology outpatient clinics in Spain, the main findings were as follows: (1) the prevalence and incidence of HF were high; (2) certain features were associated with prevalent and incident HF; and (3) the prescription of antidiabetic drugs with cardiovascular benefits was low despite suboptimal metabolic control.

This study extends the literature on the association between HF and T2D. In the Framingham study, the incidence rate of HF in T2D participants was 7.6 per 1000 person-years for men and 11.4 per 1000 person-years for women, which was 1.8 and 3.8 times higher than for their non T2D counterparts [12]. Other observational studies have confirmed this association and linked T2D to structural and functional cardiac abnormalities, such as increased left ventricular mass, left atrial enlargement, and impaired left ventricular systolic or diastolic function [18–20]. Overall, the strength of the association between HF and T2D seems to be strongly influenced by the characteristics of the analysed population, with greater risks of HF reported in those studies including elderly populations and coronary artery disease patients [21, 22]. In this regard, we found a remarkably high prevalence (39.2% at the initial visit) and incidence rate of HF (3.0 per 100 person-years) in this cohort of cardiology and endocrinology outpatient attendees, providing a solid foundation for the opportunistic screening of HF in this setting, in which HF prevalence seems particularly high. Boonman-de Winter et al. reported a prevalence of HF of 27.7% using an active comprehensive search of HF in T2D patients that also included echocardiography and natriuretic peptides assessment [23]. The slightly lower prevalence found in this study might be explained by the different characteristics of the included cohort, that was representative of a general population of T2D patients, with a lower burden of comorbidities such as coronary artery disease or CKD. In addition to age, we found an association between HF and certain disadvantageous socioeconomic factors such as a low level of education and unemployment. These adverse determinants have been previously associated with both T2D and HF, leading to an increased risk of HF, higher mortality rates and lower access to HF therapies [24–27]. The coexistence of these factors with cardiac and noncardiac comorbidities such as AF, hypertension, atherosclerotic cardiovascular disease, obesity, or CKD, should prompt clinicians to consider an underlying HF diagnosis in T2D patients [28]. In this respect, we observed a greater burden of comorbidities among HF patients in this contemporary cohort of T2D patients, highlighting the importance of a multifactorial and holistic management of these individuals. To our knowledge, there are no Spanish nationwide population-based studies detailing the characteristics of T2D patients with HF of Spain. Escobar et al. characterized a population of 21,851 HF patients from 7 regions in Spain, of whom 7371 had a T2D diagnosis [29]. The population included in the study was older (mean age 77.4 ± 10.3), with a higher proportion of women (45.4%) and a lower prevalence of coronary heart disease, which is consistent with the different healthcare setting, that not only included hospitals but also community health centres. Despite the different contexts, It should be noted than the burden of non-cardiac comorbidities was also high in this population, with 37.3% of the patients presenting CKD and 20.4% chronic obstructive pulmonary disease. In this regard, the inclusion of patients with end-stage kidney disease in this study, might have resulted in even higher rates of HF owing to the close association between the two conditions.

Despite the presence of a broad spectrum of comorbidities, NT-ProBNP has been proven to predict the risk of HF and mortality in T2D patients and is a widely available and useful tool for the early diagnosis of HF [30, 31]. In a recent consensus document of the Heart Failure Association of the European Society of Cardiology, an age-adjusted NT-ProBNP based algorithm for the diagnosis of HF in asymptomatic T2D patients was proposed [32]. In our study, we found that more than half of the patients with no baseline HF had NT-ProBNP levels greater than 125 pg/mL at the initial visit. These patients had a twofold higher risk of incident HF at 3 years. It should be acknowledged that the use of NT-ProBNP is limited in obese patients, in whom HF diagnosis might be particularly challenging due to the masking of HF related signs and symptoms [33].

Over the last decade, we have witnessed significant advancements in the pharmacological armamentarium for T2D that have led to a person-centred treatment approach that focuses not only on achieving glycaemic goals, but also on preventing and treating cardiovascular and renal comorbidities [34]. Specifically, SGLT2 inhibitors have been demonstrated to reduce the risk of heart failure hospitalization, kidney disease progression and mortality in T2D patients [35–37]. Despite the proven cardiovascular benefits of SGLT2 inhibitors and GLP-1 receptor agonists, the implementation of these therapies in our study was low, which probably explains the lack of cardioprotective effect. Less than one third of the participants receiving sodium-glucose SGLT2 inhibitors and only one in eight receiving GLP-1 receptor agonists, which might be partly related to the year of enrolment but also to other factors such as therapeutic inertia or cost containment policies on pharmaceutical expenditure within the Spanish Public Health System [38]. The gap between the benefits and the low use of these therapies in daily practice has been reported in other recent nationwide registries [39, 40]. In aggregate, the high rates of HF and the low implementation of antidiabetic drugs with cardiovascular benefits found in this cohort of T2D patients, reveal a widely untapped opportunity for HF screening, prevention, and early initiation of treatment in T2D patients visiting cardiology and endocrinology outpatient clinics.

There are several limitations of this study that should be noted. First, clinicians and high-volume hospitals willing to provide the best care for patients presenting the two conditions might be overrepresented, potentially leading to selection bias. Second, these results may not be generalizable to T2D patients in other countries with different health policies or to contemporary patients in Spain. Since the cohort was enrolled a few years ago, the implementation of T2D drugs with cardiovascular benefits was low. Higher prescription rates of these drugs might have modified our findings. Last, the outbreak of the COVID-19 pandemic during the study period reduced the number of planned visits and influenced HF admission patterns [41, 42].

## Conclusion

In this contemporary cohort of T2D patients attending cardiology and endocrinology outpatient clinics, the prevalence and incidence of HF were markedly elevated. HF was associated with unfavourable social determinants of health and additional comorbidities, particularly cardiometabolic diseases. The use of antidiabetic drugs with cardiovascular benefits was limited. Thus, outpatient cardiology and endocrinology care represent a unique opportunity for a comprehensive T2D approach that includes the diagnosis, prevention, and treatment of HF.

## Data Availability

The dataset analysed during the current study is available from the corresponding author on reasonable request.

## Abbreviations

AF: Atrial fibrillation
CKD: Chronic kidney disease
GLP1: Glucagon-like peptide-1
HF: Heart failure
LVEF: Left ventricular ejection fraction
NT-ProBNP: N-terminal pro-B-type natriuretic peptide
SGLT2: Sodium-glucose cotransporter-2
T2D: Type 2 diabetes
